# Improving Access to Cancer Care in the HIV Population: Qualitative Research to Identify Barriers to Care

**DOI:** 10.1089/heq.2020.0001

**Published:** 2020-10-30

**Authors:** Kelsey L. Corrigan, Brandon A. Knettel, Noelani Ho, Stuart Carr, Bijal Shah, Joan Cahill, Junzo Chino, Melissa H. Watt, Gita Suneja

**Affiliations:** ^1^ Duke University School of Medicine, Durham, North Carolina, USA.; ^2^ Duke Global Health Institute, Duke University, Durham, North Carolina, USA.; ^3^ Department of Population Health Sciences, Duke University Health System, Durham, North Carolina, USA.; ^4^ Department of Pediatrics Infectious Diseases, Duke University Medical Center, Durham, North Carolina, USA.; ^5^ Department of Radiation Oncology, Duke Cancer Institute, Durham, North Carolina, USA.

**Keywords:** HIV-associated cancer, health disparities, barriers to cancer care, qualitative research

## Abstract

**Purpose:** People living with HIV are less likely to receive cancer treatment and have worse cancer-specific survival, yet underlying drivers of this disparity have minimally been explored. We investigated cancer care barriers from the perspective of patients living with HIV and cancer (PLWHC) to inform future interventions, reduce disparities, and improve outcomes.

**Methods:** We conducted in-depth semistructured interviews with 27 PLWHC. The interview guide explored perceptions of the cancer care experience, treatment decision making, and barriers to cancer treatment. Interview data were analyzed using the constant comparative method of qualitative analysis.

**Results:** Study participants were predominantly men (*n*=22, 81%) with a median age of 56 years and median annual income of $24,000. Among those who experienced challenges with cancer treatment adherence, barriers included debilitating side effects of cancer treatment, stigma surrounding HIV, issues with coping and mental health, the financial burden of cancer care, and challenges with care accessibility. Despite these challenges, participants indicated that their past experiences of coping with HIV had prepared them to accept and address their cancer diagnosis. Resiliency and social support were key facilitators for cancer treatment adherence.

**Conclusion:** This qualitative study of PLWHC in the United States found that a cancer diagnosis created a substantial added stress to an already challenging situation. Health- and stigma-related stressors impacted patients' ability to fully complete cancer treatment as prescribed. There is a need for improved provider communication and mental health support for PLWHC to ensure equitable access to and completion of cancer treatment.

## Introduction

Cancer is a growing problem among people living with HIV, as several non-AIDS defining malignancies, including Hodgkin's lymphoma and lung cancer, have become more common since the widespread adoption of antiretroviral therapy (ART).^1^ Although the rise in non-AIDS defining malignancies is well documented, cancer-specific outcomes in people living with HIV are significantly worse than the general population.^2,3^ Several population-based studies have demonstrated that patients living with HIV and cancer (PLWHC) in the United States are less likely to receive cancer treatment compared with uninfected patients and that this disparity is a likely contributor to poor cancer survival.^4–6^

Published studies of the general cancer population demonstrate that patients who are older, of ethnic or racial minorities, or are uninsured/underinsured are less likely to receive cancer treatment.^7–9^ The underlying cancer treatment disparities in PLWHC are likely driven by similar social and health systems factors; however, the additive burden of living with a chronic potentially life-threatening illness may pose additional challenges.^4–6^ Physician-prescribing behavior may play a role as a survey study showed that oncologists were less likely to offer standard cancer treatment to PLWHC if they believed treatment toxicity was great and efficacy was lower.^10^ Analogous studies of cancer treatment disparities from the patient's perspective are limited.

A greater understanding of barriers to cancer therapy in PLWHC is urgently needed to develop strategies to improve access to cancer care and enhance treatment completion for these patients. To address this gap in the literature, we conducted qualitative interviews with PLWHC to understand their perceptions of the cancer care experience.

## Methods

We completed qualitative interviews with 27 patients living with HIV who were diagnosed with cancer. Interviews were conducted between December 2017 and January 2019. The study received ethical approval from the Institutional Review Board at Duke University. The study was performed at Duke University Medical Center, which has an HIV clinic serving ∼2,000 patients and a cancer center serving ∼70,000 patients annually. The demographics of HIV patients served by the medical center largely mirror the U.S. HIV population, with a majority of patients who are men, African American, and with low income.^11^

### Sample and recruitment

We used DEDUCE software and electronic medical record (EMR) review to identify patients living with HIV who were diagnosed with cancer after HIV infection. To capture a variety of perspectives in our study population, we enrolled patients from different stages in the care process, including those who had a history of cancer with completed treatment, were recently diagnosed with cancer and currently receiving treatment, or had a past or active cancer diagnosis and experienced challenges with cancer care. An equal number of patients corresponding to these categories were enrolled. Cancer care challenges were defined as missed appointments or early discontinuation of treatment, and were determined by EMR review. Patients <18 years old were excluded from the study.

At the patient's next clinic appointment, we introduced the study, obtained written informed consent, and scheduled the interview date. Interviews lasted approximately 60–90 min. After the interview, a $60 cash remuneration was provided to patients.

### Qualitative interview guide

The research team developed the semistructured interview guide from prior literature on cancer in patients living with HIV and three relevant theoretical frameworks: the Health Belief Model, PEN-3 Model, and Anderson Healthcare Utilization Model.^12–14^ Questions and prompts explored perceptions regarding the cancer care experience, cancer treatment decisions, and facilitators of and barriers to cancer treatment.

### Analysis

Audio-recorded interviews were transcribed verbatim. The constant comparative method of qualitative analysis was used to identify emerging patterns and perceptions from the data that could be operationalized into concrete themes (Table 1).^15^ Individual memos were written for each transcript to summarize the content and highlight emerging themes.^16^ Memos and emerging themes were shared in research team meetings to build consensus on the coding structure. Guided by the team discussions and theoretical frameworks of the study, data were then integrated into a structured codebook in NVivo 12 software. Team meetings also iteratively explored the topic of data saturation, and study enrollment was discontinued once saturation was reached. A subset of nine interviews, three from patients with a past cancer diagnosis, three from patients with an active cancer diagnosis, and three from patients with cancer care challenges noted in the EMR, were randomly selected to be recoded by a second team member and checked for intercoder agreement. Coding differences were reconciled for consensus. A pre-established threshold of 80% intercoder agreement was achieved.^17^ Finally, coded texts were reviewed and synthesized, leading to consensus-building discussions about the emerging themes. Topic and method experts reviewed the final themes and conceptual models.

**Table 1.** Constant Comparative Method Study Design

Stages	Study design
(1) Compare incidents applicable to each category	Code participant responses by category into qualitative memos
(2) Integrate categories and their properties	Inductively develop codebook, identify patterns, reconcile differences
(3) Delimit the theory	Finalize the codebook and code memos
(4) Write the theory	Identify and synthesize broader themes based on coding

*Source:* Glaser.^15^

## Results

### Participant characteristics

The study sample included 22 (81.5%) men and 5 (18.5%) women with a median age of 56 years and a median annual household income of $24,000. Nineteen (70.4%) were African American, seven (25.9%) were Caucasian, and one (3.7%) was Hispanic/Latino. Eleven (40.7%) participants held a high school diploma or less. Eight (29.6%) participants had private insurance, while the remainder had Medicare, Medicaid, or were uninsured. Participants were heterogeneous in their cancer type and stage. Participant clinical characteristics are summarized in Table 2.

**Table 2.** Participant Clinical Characteristics (*n*=27)

	Past cancer group, n (%)	Active cancer group, n (%)	Treatment challenges group, n (%)	All participants, N (%)
Total	9 (33.3)	9 (33.3)	9 (33.3)	27 (100.0)
CD4 count at time of cancer diagnosis				
Median	345	234	287	301
Viral load at time of cancer diagnosis				
Undetectable	8 (88.9)	4 (44.4)	4 (44.4)	16 (59.3)
20–100 copies/mL	0	1 (11.1)	2 (22.2)	3 (11.1)
100–1,000 copies/mL	0	0	2 (22.2)	2 (7.4)
>1,000 copies/mL	0	1 (11.1)	1 (11.1)	2 (7.4)
Unknown	1 (11.1)	3 (33.3)	0	4 (14.8)
Time between HIV and cancer diagnosis				
Median, years	18	4	15	14
Cancer site				
Anus	3 (27.3)	1 (8.3)	5 (38.5)	9 (25.0)
Non-Hodgkin's lymphoma	1 (9.1)	4 (33.3)	0	5 (13.9)
Hodgkin's lymphoma	2 (22.2)	0	2 (15.4)	4 (11.1)
Prostate	1 (9.1)	1 (8.3)	2 (15.4)	4 (11.1)
Head and neck	1 (9.1)	2 (16.7)	1 (7.7)	4 (11.1)
Colorectum	2 (22.2)	0	0	2 (5.6)
Liver	0	2 (16.7)	0	2 (5.6)
Vulva	0	0	2 (15.4)	2 (5.6)
Lung	0	1 (8.3)	0	1 (2.8)
Bladder	0	1 (8.3)	0	1 (2.8)
Thyroid	1 (9.1)	0	0	1 (2.8)
Penis	0	0	1 (7.7)	1 (2.8)
Cancer stage				
Stage I	5 (55.6)	0	2 (22.2)	7 (25.9)
Stage II	0	0	3 (33.3)	3 (11.1)
Stage III	3 (33.3)	1 (11.1)	2 (22.2)	6 (22.2)
Stage IV	1 (11.1)	6 (66.7)	2 (22.2)	9 (33.3)
Unknown	0	2 (22.2)	0	2 (7.4)

### Satisfaction with cancer care

Participants described their reactions to a cancer diagnosis as being similar to their initial reactions to their HIV diagnosis, characterized by feelings of shock, fear, and hopelessness: “No one wants to hear that word cancer, that you have it … Now you have this diagnosis and the first thing you think is okay, death is next. Life is almost over.”

Despite the weight and impact of receiving a cancer diagnosis, the majority of participants were generally satisfied with their providers, describing the demeanor of providers as kind, supportive, and appropriately concerned when relaying the cancer diagnosis. Participants reported that their providers were knowledgeable, competent, and “well-known experts within the field.” Participants described similar positive experiences with nurses and other hospital staff throughout treatment and were satisfied with the cancer care they had received overall.

### Communication with providers during cancer treatment

Although most participants described being satisfied with their care, those unsatisfied with their care noted lapses in communication with their providers. Participants with cancer care challenges evident in the EMR described negative care experiences more often than participants without challenges noted in the EMR. The most common communication challenge was not receiving sufficient information from providers about treatment. This included feelings that they were inadequately prepared for treatment side effects, or that information about cancer was delivered with a “poor bedside manner.” As one participant recalled: “I wished they would have told me a little bit more [about what to expect from treatment]. I still would've done the radiation, but I didn't realize how debilitating it would be.”

Participants also expressed concern about the potential impact of cancer treatment on their HIV, but there was wide variation in the extent to which HIV was discussed with cancer providers. About one-third of participants said HIV was never discussed. Several participants reported that the burden fell on them to intiate conversations about HIV and cancer: “I mean, they answer the questions…but you have to ask it.”

### Coordination among cancer and HIV providers

About half of participants perceived that there was active communication between their cancer and HIV providers. The other half perceived a lack of communication, passive communication through medical records, or were unaware of whether or not there was communication among providers. Some participants expressed dissatisfaction with the lack of communication among providers: “I would say that HIV providers need to be more involved with the oncologist … I would like for them to verbally talk, versus just reading medical records.” Those who did report active communication indicated that they were confident that their providers were regularly communicating and that they viewed the coordination positively: “I told [the oncologist and Infectious Disease physician], I need you to be on the same team, not separate. I made sure they both were communicating.”

### Cancer treatment adherence

Study participants with a cancer history, active cancer diagnosis, and cancer care challenges noted in the EMR all described barriers related to cancer care (Table 3). Sometimes, these challenges affected their ability to complete cancer treatment, with treatment side effects and treatment fatigue cited as the most important barriers to care. One participant recalled: “I couldn't move my bowels and was throwing up and coudn't eat … I was hurting and I couldn't, I just said no, I'm not doing it no more and I stopped.” Other participants struggled with adherence due to emotional and mental health challenges; as one participant described, “I get weak, don't get me wrong. I break down all the time. I cry every day. You may not see it, somebody may not see it, but I do.” Other causes of adherence challenges included poor experiences with providers and accumulated external stressors: “And then my sister passed away, and I stopped everything except my AIDS medicine because I was overwhelmed … I stopped everything. I gave up.”

**Table 3.** Summary of Qualitative Themes Related to Barriers to Cancer Care

Barriers to cancer care	Frequency, N (%)	Illustrative quotation
Side effects from treatment	24 (88.9)	“I had terrible side effects … I got to the hospital, got out my truck and that was as far as I made it … I fell down and I crawled from my truck to the hospital.”
Stigma	18 (66.7)	“[The nurse] was so nice and provided great care. Then one day she just stopped coming. She went on the computer and saw my [HIV] status and never came back.”
Accessibility issues^a^	15 (55.6)	“[Parking] has just been an issue that you deal with. It's pretty brutal to go over to [the clinic], find a parking place, park, and get to your appointment on time.”
Financial burden of cancer treatment	14 (51.9)	“When I was diagnosed [with cancer], it put a big financial burden on me because I wasn't able to go to work. Thank God my family helped me out on my mortgage so I wouldn't lose my home. But I'm still trying to recover from some of that. And some stuff you probably will never recover from.”
Emotional and mental health difficulties	14 (51.9)	“I understand why people do suicide. I get it cause you're tired of fighting. … You know I've been there, where you're just tired of pain, you have no hope, you don't see anything over the horizon and just want rest. You just want it to be over.”
Family or personal issues	4 (14.8)	“My sister had an aneurysm and just drops dead … Then, I said, I can't do [the cancer treatments] anymore … I'm hurting and I couldn't, I just said, no, I'm not doing it no more. I stopped everything and I gave up.”
Mistrust in providers	4 (14.8)	“When you have a doctor that's iffy, it's an entirely different thing altogether because you're not sure, you're never sure of whether he's right or wrong.”
Fear about cancer diagnosis and treatments	3 (11.1)	“Fear held me back [from getting care], … fear and poor self-worth and isolation and degradation and things like that.”

^a^ Accessibility issues include transportation difficulties (long drive, parking) and long wait times.

Among participants who completed cancer treatment, several common themes emerged regarding their motivations: a commitment to being cured, trust in their care team, and positive relationships with their providers. These participants typically described having complete confidence in their providers' advice. Other participants attributed their adherence to their families, citing strong familial support in coping with their diagnosis, attending appointments, and persevering through the challenges of treatment.

### Financial burden of HIV and cancer

Participants described the impact of HIV and cancer diagnoses on their financial situation, with those with cancer care challenges described in the EMR reporting the greatest amount of financial stress. Many participants described being unable to work due to physical side effects and the emotional burden of cancer treatment: “I can't work. When I get in an argument with a customer, I just cry. I feel like [my cancer] is turning me into a bad person because I lash out. It's only the illness, it's not me.”

Among those who were able to continue working, several said that they frequently had to take days off to attend treatment appointments, which impacted their income. Several participants also noted there were costs associated with attending appointments, including transportation and parking. For many of these challenges, they relied on family and friends to assist with direct financial support, which was very valuable in helping them cope with their treatment.

### Stigma among support system and providers

When asked to describe the support they had received throughout their cancer treatment, most participants cited partners, family members, and close friends as invaluable in providing emotional and logistical support. However, several participants described fear of stigma from loved ones, particularly related to HIV, and how this fear had prevented them from disclosing their HIV status or seeking support. This fear deterred these participants from involving their loved ones in their cancer care, leaving them without a critical source of advocacy and support during their cancer appointments and treatments. Stigma was also perceived in the health care setting. Although infrequent, some participants noted that health care providers treated them differently because of their HIV diagnosis. One participant described that “[healthcare providers] get geared up, which they don't do with other patients. I was just like, um, are you scared? You think I'm going to contaminate you?”

Other impactful forms of stigma occurred surrounding participants' sexuality and race. A few participants felt isolated as a result of the overlapping stigmas related to their HIV, sexuality, and/or race, and wished they had more support. Some said these intersecting stigmas were particularly difficult to cope with after their cancer diagnosis due to the accumulative burden of their stressors: “I used to go to a support group here [for people with HIV], but then they stopped it. I miss it … Since then I have just been a private person.”

### Mental health treatment

About one-third of participants reported past or current symptoms of depression and anxiety. In discussing the stressors that contributed to these symptoms, participants often referred to their dual diagnosis of HIV and cancer, their experience with cancer treatments, or the painful side effects of cancer treatment that they endured: “I have been known to go through bouts of depression when [side effects] get really bad. I wanted to die the first time because it was just so painful and miserable.”

Several participants reported experiencing suicidal ideation, most commonly after their HIV diagnosis or at some point during their cancer treatment: “I think I was more devastated with the HIV, ‘cause when I found out I had HIV, I wanted to die. I was thinking about all these ways I could kill myself.”

Of those who received mental health treatment, about half reported using pharmacological treatment and half utilized therapy. Most participants who saw a therapist found it to be helpful. However, a few participants explained that they could no longer afford their psychiatric medications or therapy, which posed a significant obstacle to controlling their mood disorders: “Now I'm going to tell you, if insurance paid for it I'd go back to therapy. I loved it. It's [helped me to be] more centered and it changed the way you looked at things.”

### Resilience

After their cancer diagnosis, more than half of the study participants reported that their earlier experiences with HIV had provided them with emotional tools, strength, and resiliency to more effectively cope with their cancer treatment: “I realized that the HIV wasn't going to kill me and I began to live my life. When I found out I had cancer, I was like well I've lived this long so I'm gonna beat this too.”

The resiliency stemming from their initial HIV diagnosis inspired many participants to have a positive attitude and seek support to help them complete their cancer treatment. A majority of participants cited family as an essential aspect to their resiliency and their motivation to seek and complete cancer treatment: “What motivated me was to see my children grow … When faced with a deadline, with that period at the end of your sentence, you start to embrace life more. Until somebody tells you you're gonna die, you don't really live.”

## Discussion

In this study of individuals living with HIV and cancer, the most commonly described cancer care barriers were cancer treatment side effects, HIV stigma, accessibility issues, costs of care, and emotional and mental health difficulties. Despite these challenges, participants shared stories of resiliency and strong social support, which aided them in their adherence to cancer care and motivated them to complete treatment.

The most commonly described barrier to care was cancer treatment side effects. The impact of cancer treatment side effects has been described in prior studies.^18–20^ In PLWHC, the impact of cancer treatment side effects on HIV treatment adherence must also be considered. Although there is limited literature describing adherence to HIV treatment among PLWHC, both oncologists and infectious disease specialists should monitor cancer treatment side effects and ensure that both cancer and antiretroviral therapies are optimized to minimize treatment toxicity.

Communication between physicians and patients was a frequently discussed topic in our qualitative interviews. Open and honest communication between patients and providers is essential for treatment completion for acute and chronic diseases.^21^ In this study, we found that many participants were unsatisfied with provider communication because they felt they were given insufficient information about treatment side effects and how their cancer treatment would affect their HIV. Moreover, participants who experienced challenges with cancer treatment completion cited poor communication more often. Communication about the cancer treatment process and potential side effects specific to HIV may play a key role in preparing patients for their treatments and motivating them to stay engaged in care.

Many participants in this study expressed that they were confident their oncologist and HIV provider communicated on a regular basis. However, a prior study surveying U.S. oncologists about their care of PLWHC found that active collaboration between the two types of providers was very limited.^10^ Collaboration among oncologists and HIV providers has the potential to improve provider confidence in patient management, minimize overlap in toxicities between cancer treatment and ART, and reduce treatment disparities in PLWHC.

Stigma was another frequently discussed barrier that affected study participants' cancer care. Several other studies have corroborated the role of enacted or anticipated HIV stigma as negatively affecting treatment adherence.^22–24^ Given advances in ART and longer life expectancies among patients living with HIV, providers must recognize their personal biases about PLWHC and how these may influence care decisions. Providers who initiate affirming and patient-centered conversations about HIV during treatment may pre-empt patient fears about stigma, which can lead to improved care engagement.^25^ Mitigating implicit bias and disbanding the overlapping stigma in PLWHC are important steps in reducing disparities in this population.^26,27^

Past studies examining disparities in treatment outcomes for PLWHC have focused on patient demographics as potential predictors of not receiving cancer treatment. However, our findings indicate that these patients face additional barriers that are difficult to capture in registry studies, but which may interfere with the ability to initiate and/or complete cancer treatment. These barriers, such as symptom burden, mental health symptoms, frequency of appointments, and cost of care, are simultaneously related to both cancer and HIV, presenting a cumulative burden to PLWHC.^28^ There is a need for larger studies to assess the generalizability and relative impact of patient-level factors described in our study and to inform efforts to improve cancer outcomes among PLWHC.

The results of this study should be interpreted in light of its limitations. All participants were recruited from a single, large academic medical center in one U.S. city with high HIV prevalence. Patient perspectives would likely differ in smaller health systems or in settings with fewer resources. Also, the generalizability of the findings is limited given the small sample size of the study. Although we assured participants that their interview responses would in no way impact their care, it is possible that social desirability influenced participant responses. Finally, we focused only on soliciting patient perspectives in this study. The results are intended to build upon previous research examining provider perspectives about care among people with HIV and cancer,^10^ and should be interpreted in the context of those results.

## Conclusion

This is the first qualitative study to describe the perspectives of PLWHC in the United States. Many HIV-related factors, including stigma, cost of care, and mental health difficulties, created barriers to cancer treatment on top of already challenging aspects, such as side effects and accessibility issues. This study highlights the opportunity to improve the comfort and skill of cancer providers to discuss HIV with patients and liase with HIV care physicians. In addition, there should be greater mental health support for PLWHC to aid with management of this complex patient population. There is an urgent need to develop strategies that address barriers, reduce care disparities, and give PLWHC equitable access to crucial cancer treatment.

## Abbreviations Used

ART: antiretroviral therapy
CFAR: Center for AIDS Research
EMR: electronic medical record
PLWHC: patients living with HIV and cancer
